# Recent Advances in Glucose Biosensors

**DOI:** 10.3390/bios16040222

**Published:** 2026-04-16

**Authors:** Natalija German, Anton Popov

**Affiliations:** 1 Department of Immunology and Bioelectrochemistry, State Research Institute Centre for Innovative Medicine, Santariskiu 5, LT-08406 Vilnius, Lithuania; 2 NanoTechnas—Center of Nanotechnology and Materials Science, Institute of Chemistry, Faculty of Chemistry and Geosciences, Vilnius University, LT-03225 Vilnius, Lithuania

## 1. Introduction

The global burden of diabetes continues to grow, and the disease ranks among the leading causes of death and disability worldwide; it is projected that by 2050, the number of people living with this disease will exceed 1.3 billion. Effective blood glucose level monitoring is crucial for preventing and treating diabetes, as well as for maintaining overall well-being. Due to their widespread use and practical advantages, especially for continuous glucose monitoring (CGM), electrochemical glucose biosensors are expected to account for more than 85% of the global biosensor market [1]. Such biosensors consist of a biorecognition element and an electrochemical transducer and can be easily miniaturized and applied for CGM. An electrochemical glucose biosensor is among the most successful point-of-care (POC) devices.

Enzymatic glucose biosensors represent a major segment of the global market. Glucose oxidase (GOx) and horseradish peroxidase (HRP) are commonly used in their fabrication. In such devices, GOx-catalyzed glucose oxidation generates hydrogen peroxide (H_2_O_2_) and changes in oxygen (O_2_) concentration, and these signals are proportional to glucose concentration. Moreover, the incorporation of electron transfer mediators enhances the performance of the biosensor by eliminating the dependence of the analytical signal on the O_2_ concentration. An alternative strategy involves combining HRP with GOx, where HRP catalyzes the reduction in H_2_O_2_. In such systems, electron transfer may occur between the HRP active site and the electrode surface, whereas effective direct electron transfer is very challenging for GOx. Despite widespread distribution of enzyme-based biosensors, their limitations, such as low thermal and chemical stability, have driven the search for other options. Efforts to overcome these limitations commonly focus on improving enzymatic biosensors through the integration of new engineering and technological solutions, nanostructures, and algorithmic and artificial intelligence (AI)-based approaches. At the same time, the non-enzymatic biosensors are attracting considerable attention as a promising alternative platform [2]. However, most proposed solutions are still at the prototype or near-application stage, where the design of POC devices and wearable CGM systems is actively under development. A key motivation behind these efforts has been the development of convenient, painless, noninvasive, and highly accurate glucose monitoring devices [3].

Comprising diverse contributions, the Special Issue “Recent Advances in Glucose Biosensors” offers both innovative original research and in-depth review perspectives in glucose biosensing. The Special Issue highlights various advancement strategies of glucose biosensors and examples of their real-world application.

## 2. Overview of Contributions

This Special Issue presents nine articles that, together, map a diverse electrochemical glucose biosensor landscape and delve into key topics in this field. For clarity, the contributions are arranged into three thematic sections: (i) Non-Invasive, Wearable, and Minimally Invasive Glucose Sensing; (ii) Nanomaterial-Based Electrochemical Platforms and Energy-Integrated Glucose Biosensors; and (iii) Foundational Perspectives, Modeling, and Clinical Context. Each is briefly introduced with respect to its target, methodological approach, and the advances they report.

### 2.1. Non-Invasive, Wearable, and Minimally Invasive Glucose Sensing

CGM is of significant clinical importance. Microneedles (MNs) technology offers a minimally invasive method for detecting glucose levels in interstitial fluid. Currently, scientists and engineers are conducting intensive research to improve this technology towards commercial CGM systems. Choi et al. (contribution 1) proposed an analytical system based on an array in which GOx was immobilized on gold-coated MNs through a 3-mercaptopropionic acid self-assembly monolayer using carbodiimide chemistry. The system demonstrated reliable performance by measuring impedance as a function of glucose level in phosphate-buffered solution for up to ~10 days. Despite the need for further technology development, the shadow mask used to fabricate the MNs array offers significantly lower production costs than the standard photolithography techniques. Skonta et al. (contribution 2) reported the 3D printing of MNs using polylactic acid (PLA) filament. The MNs were loaded with chitosan nanoparticles with embedded GOx, HRP, and 2,2′-azino-bis(3-ethylbenzothiazoline-6-sulfonic acid) (ABTS) as a chromogenic substrate. Fabricated MNs patch arrays enabled colorimetric detection of glucose levels in sweat samples. The color change from glucose and ABTS oxidation, driven by the cascade enzymatic reaction of GOx and HRP, was evaluated using AI to study smartphone photographs. Such a system was able to detect glucose in 5 µL of sample over a linear range of 0.025 to 0.375 mM, with a limit of detection (LOD) of 0.023 mM.

### 2.2. Nanomaterial-Based Electrochemical Platforms and Energy-Integrated Glucose Biosensors

Nanomaterials are actively used to enhance the analytical performance of electrochemical glucose biosensors. Four studies in this Special Issue describe the application of nanomaterials in various ways. Ahmed et al. (contribution 3) report the integration of carbon nanotubes (CNTs) with MnO_2_ nanorods (NRs) to form a nanocomposite for the fabrication of a nonenzymatic glucose biosensor. Biosensors based on a freestanding electrode, fabricated by depositing the nanocomposite on carbon paper, demonstrated good catalytic activity toward glucose oxidation over a linear range of 0.5–10 mM, with a sensitivity of 309.73 µA cm^−2^ mM^−1^ and LOD of 0.19 mM. Improvement in biosensor analytical parameters achieved by adding CNTs to MnO_2_—NRs demonstrated the promising potential of these nanostructures for biosensing.

Despite the advantages of nonenzymatic biosensors, enzymatic biosensors still occupy a large share of the commercial glucose biosensor market. The incorporation of nanomaterials can enhance the strengths of such biosensors while mitigating their inherent limitations. German et al. (contribution 4) reported the application of a graphite rod (GR) modified with dendritic gold nanostructures (DGNS) and a cystamine (Cys) self-assembled monolayer (GR/DGNS/Cys) as a platform for working electrode fabrication. The GOx was immobilized either directly onto the GR/DGNS/Cys electrode (1st electrode) or onto the same electrode after pre-modification with a nanocomposite of polyaniline (PANI) and gold nanoparticles (AuNPs) (2nd electrode), synthesized enzymatically using GOx. Proposed reagentless biosensors exhibit similar sensitivity (1st—93.7 and 2nd—72.0 µA mM^−1^ cm^−2^) and LOD (1st—27 and 2nd—34 µM), as well as exceptional 71-day storage stability, while PANI-AuNPs nanoconjugate improved the glucose detection in the serum. Kausaite-Minkstimiene et al. (contribution 5) proposed the application of bimetallic platinum-cobalt (PtCo) as a nanozyme, possessing peroxidase-like catalytic properties, for the fabrication of a reagentless glucose biosensor. The GR electrode was modified layer-by-layer with PtCo, Nafion, and GOx. Glucose detection was based on the electrocatalysis of H_2_O_2_ generated during the enzymatic GOx reaction by PtCo. The fabricated biosensor detected glucose levels in the linear range from 0.04 to 2.18 mM, with a sensitivity of 19.38 μA mM^−1^ cm^−2^ and LOD of 21 µM.

Another direction in the design of glucose biosensors is the use of an enzymatic biofuel cell (EBFC). Kausaite-Minkstimiene et al. (contribution 6) modified the cathode with Prussian blue nanoparticles embedded in poly(pyrrole-2-carboxylic acid) (PPCA), while the anode was modified with a nanocomposite of poly(1,10-phenantroline-5,6-dione) and AuNPs in a PPCA shell. GOx was covalently immobilized on both electrodes. With these EBFC electrode modifications, the team achieved a sensitivity of 0.16 μA mM^−1^, LOD of 0.07 mM, and a wide linear range of 0.15–124.00 mM. Moreover, good reproducibility, selectivity, and operational stability over 35 days were demonstrated, and the biosensor was suitable for measurements in serum. Although EBFC-based glucose detection is still at an early laboratory stage, this study shows the potential of such technology.

### 2.3. Foundational Perspectives, Modeling, and Clinical Context

Several studies described biosensor fabrication, the need for validation, and highlighted the importance of assessing device real-world applications. Rajamohan et al. (contribution 7) reviewed the role of various nanostructures in glucose biosensing. They presented the evolution of glucose biosensors, followed by the application of categorized nanostructures in the fabrication of electrochemical, optical, and wearable biosensors. This review highlights the importance of integrating nanotechnology and microfluidic devices to advance glucose biosensing innovation. Pedro et al. (contribution 8) emphasized the importance of validating analytical models for optical glucose detection methods. They measured serum absorption and found that absorption at 975 nm depends on glucose concentration, highlighting the need for further studies to evaluate the impact of various serum compounds on the proposed analytical model and to develop non-invasive glucose sensing technologies.

The CGM is an important component of type 1 diabetes (T1D) management. However, effective systematic diabetes monitoring in daily life requires a degree of independent glycemic control, which can be challenging for adolescents. Kietaibl et al. (contribution 9) showed that a one-week adventure camp for adolescents (12–18 years) with T1D, organized without a structured educational protocol and with minimal diabetes-specific training, was feasible and safe. Participants used a standardized real-time GCM system (Dexcom sensor). Glycemic control improved during the camp compared with baseline, but the effect was not sustained after the camp. The findings support the importance of the preparation and training process for successful real-world GCM use.

## 3. Conclusions and Outlooks

Nowadays, glycemic control is driving the miniaturization of electrochemical glucose biosensors, enabling the fabrication of wearable, portable, flexible, and easily operated devices. Such an approach leads to the decentralization of the POC testing system, revolutionizing diagnostics and the health care system [4]. The evolution of technologies such as mobile devices, cloud services, and wireless communication, which enable large-scale data transfer, allows real-time health management. However, the development of biosensors must go hand in hand with these technological advances.

The evolution of electrochemical biosensors requires movement in multiple directions to combine innovation into a single digital device. The integration of nanostructures enhances thermal and chemical stability, as well as sensitivity and selectivity. Extending the lifespan of glucose biosensors remains one of the most critical challenges and may be addressed by identifying suitable nanomaterials that improve their lifespan.

At present, glucose detection in interstitial fluid (ISF) is considered one of the most promising minimally invasive approaches for continuous monitoring. In contrast, the correlation between glucose levels in sweat, saliva, and tears and those in blood remains insufficiently understood. Nevertheless, glucose detection in ISF still presents significant challenges [3]. The development of microneedles and similar technologies reflects the ongoing search for new materials and their integration with advances in microfluidic devices.

Technological solutions are necessary to minimize drift over time, to refine glucose biosensor calibration, and especially to improve calibration stability. The search for a new calibration algorithm and the development of self-calibrating protocols are at the forefront of research. The integration of artificial intelligence can improve the prognostic assessment of potential hypoglycemic or hyperglycemic events [5]. Moreover, glucose detection as a part of multiplex sensing is seen as the future of POC devices.

By bringing together advances in sensor materials, device design, and digital integration, this Special Issue “Recent Advances in Glucose Biosensors” demonstrates the broad potential of glucose biosensors to become practical and effective tools for modern diabetes diagnosis and treatment.

## Data Availability

The raw data supporting the conclusions of this article will be made available by the authors on request.
